# Supplementary figures and images for: Cerebral Metabolic Rate of Glucose and Cognitive Tests in Long COVID Patients
Source: Brain Sci. 2022 Dec 22;13(1):23. doi: 10.3390/brainsci13010023 (PMC9856023; doi:10.3390/brainsci13010023)

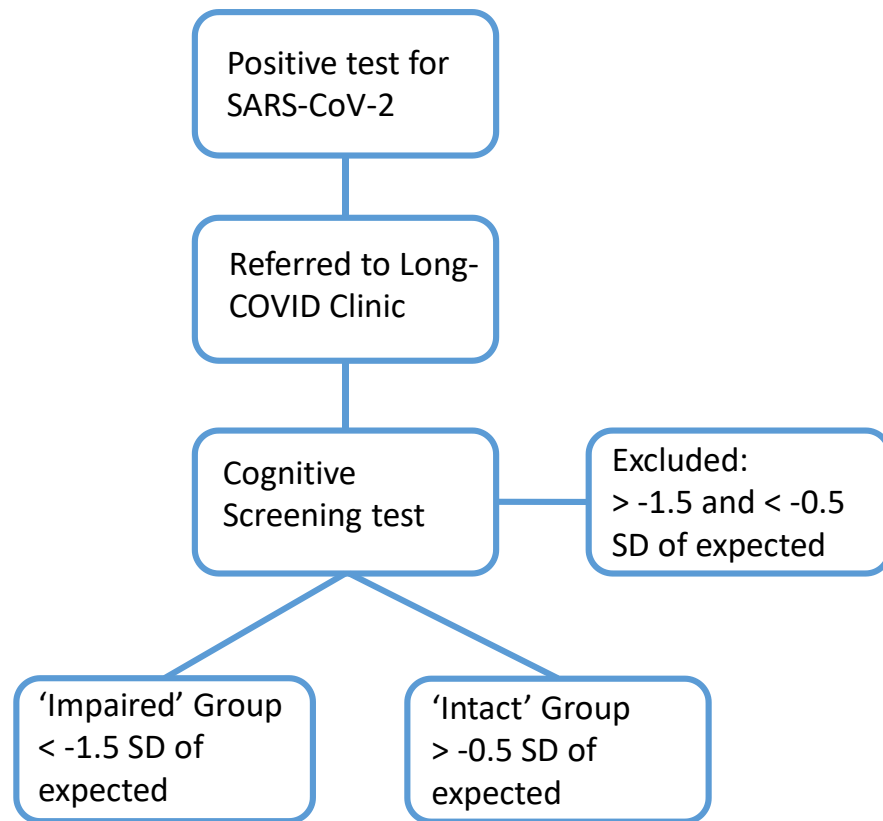

Figure S1: Schematic diagram of inclusion.

Supplement: Supplementary file 1 [file brainsci-13-00023-s001.zip › brainsci-2073895-supplementary.pdf]
